# Molecular landscape of *TP53* mutations in breast cancer and their utility for predicting the response to HER‐targeted therapy in HER2 amplification‐positive and HER2 mutation‐positive amplification‐negative patients

**DOI:** 10.1002/cam4.4652

**Published:** 2022-04-07

**Authors:** Binliang Liu, Zongbi Yi, Yanfang Guan, Quchang Ouyang, Chunxiao Li, Xiuwen Guan, Dan Lv, Lixi Li, Jingtong Zhai, Haili Qian, Binghe Xu, Fei Ma, Yixin Zeng

**Affiliations:** ^1^ Department of Medical Oncology, National Cancer Center/National Clinical Research Center for Cancer/Cancer Hospital Chinese Academy of Medical Sciences and Peking Union Medical College Beijing China; ^2^ Department of Breast Cancer Medical Oncology, Hunan Cancer Hospital and the Affiliated Cancer Hospital of Xiangya Medical School Central South University Changsha Hunan China; ^3^ Geneplus‐Beijing Institute Beijing China; ^4^ Department of Computer Science and Technology, School of Electronic and Information Engineering Xi'an Jiaotong University Xi'an Shaanxi China; ^5^ State Key Laboratory of Molecular Oncology, National Cancer Center/National Clinical Research Center for Cancer/Cancer Hospital Chinese Academy of Medical Sciences and Peking Union Medical College Beijing China; ^6^ Chinese Academy of Medical Sciences and Peking Union Medical College Beijing China; ^7^ Sun Yat‐sen University Cancer Center, State Key Laboratory of Oncology in South China Collaborative Innovation Center for Cancer Medicine Guangzhou China

**Keywords:** anti‐HER2 treatment, breast neoplasms, circulating tumor DNA, mutation, next‐generation sequencing, *TP53*

## Abstract

**Purpose:**

We used targeted capture sequencing to analyze *TP53*‐mutated circulating tumor DNA (ctDNA) in metastatic breast cancer patients and to determine whether *TP53* mutation has predictive value for anti‐human epidermal growth factor receptor 2 (HER2) treatment for in HER2 amplification‐positive patients (HER2+) and HER2 mutation‐positive, amplification‐negative (HER2−/mut) patients.

**Patients and Methods:**

*TP53* mutation features were analyzed in the Geneplus cohort (n = 1184). The MSK‐BREAST cohort was used to explore the value of *TP53* mutation in predicting anti‐HER‐2 antibody efficacy. Sequencing of ctDNA in phase Ib, phase Ic, phase II clinical trials of pyrotinib (HER2+ patients), and an investigator‐initiated phase II study of pyrotinib (HER2−/mut patients) were performed to analyze the relationships between *TP53* mutation and prognosis for HER2 TKIs. The MSK‐BREAST cohort, MutHER, and SUMMIT cohort were used for verification.

**Results:**

*TP53* mutations were detected in 53.1% (629/1184) of patients in the Geneplus cohort. The *TP53* mutation rate was higher in HR‐negative (*p* < 0.001) and HER2 amplification‐positive (*p* = 0.015) patients. Among patients receiving anti‐HER2 antibody therapy, those whose tumors carried *TP53* mutations had a shorter PFS (*p* = 0.004). However, the value of *TP53* mutation in predicting HER2 TKI response was inconsistent. In HER2+ patients, no difference in PFS was observed among patients with different *TP53* statuses in the combined analysis of the pyrotinib phase Ib, phase Ic, and phase II clinical trials (*p* = 1.00) or in the MSK‐BREAST cohort (*p* = 0.62). In HER2−/mut patients, *TP53* mutation‐positive patients exhibited a trend toward worse prognosis with anti‐HER2 TKI treatment than *TP53*‐wild‐type patients in our investigator‐initiated phase II study (*p* = 0.15), and this trend was confirmed in the combined analysis of the MutHER and SUMMIT cohorts (*p* = 0.01).

**Conclusions:**

*TP53* mutation can be used to identify biomarkers of anti‐HER2 antibody drug resistance in HER2+ patients and HER2 TKI resistance in HER2−/mut patients.

## Graphical abstract

Tp53 is one of the most common mutations in cancer patients. In this study, we first studied the mutation spectrum of Tp53 from a large cohort of breast cancer. Through the ctDNA detection and analysis of four clinical trials of pyrotinib lead by our center, and combined with the public database to verify their conclusions, we found that Tp53 can be used as a biomarker for anti‐HER2 treatment strategy selection. Tp53 gene mutation can be used to identify biomarkers of anti‐HER2 antibody drug resistance in HER2 amplification‐positive patients and HER2 TKI resistance in HER2 mutation‐positive and amplification‐negative patients.

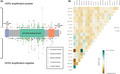

## 1 INTRODUCTION

Breast cancer is the most common malignancy in women worldwide, and 15%–20% of patients have human epidermal growth factor receptor 2 (HER2) amplification.^1^ This subgroup of patients with HER2 amplification (HER2+) can benefit from HER2‐targeted therapy. Anti‐HER2 antibody drugs such as trastuzumab and pertuzumab, small‐molecule tyrosine kinase inhibitors (TKIs) such as lapatinib, neratinib, and pyrotinib, and antibody‐drug conjugates such as trastuzumab deruxtecan (DS‐8201) and trastuzumab emtansine (T‐DM1) also show good curative effects in these patients.

For patients who have HER2 mutations without amplification (HER2−/mut), research in recent years has suggested that the irreversible pan‐HER TKI drugs neratinib (MutHER study^2^ and SUMMIT study^3^) and pyrotinib^4^ are effective and have acceptable side effects. Somatic mutations in HER2 are detected in approximately 2%–5% of primary breast cancer patients, mostly as HER2 nonamplification,^4^ and treatment with neratinib or pyrotinib is a suitable option for this population.

However, not all HER2+ or HER2−/mut patients respond equally to anti‐HER2 treatment. Therefore, the search for biomarkers that reliably predict the efficacy of anti‐HER2 therapy is important to assist physicians in the selection of precise HER2‐targeted therapies.

*TP53* is one of the most commonly mutated genes in breast cancer, and it has been reported that up to 30%–40% of breast cancer patients carry *TP53* mutations.^5^, ^6^, ^7^ Nevertheless, the value of *TP53* in predicting the efficacy of anti‐HER‐2 therapy remains unclear. Therefore, we carried out this study to explore *TP53* as a candidate biomarker of anti‐HER2 therapy response. We first used the Geneplus cohort due to its large sample size to derive preliminary data. We explored the mutation spectrum of the potential candidate marker *TP53* and the value of such mutations in predicting anti‐HER2 treatment response. We further integrated the results of four clinical studies of pyrotinib conducted at our center and verified our findings using three external datasets (Memorial Sloan Kettering Cancer Center (MSK)‐BREAST,^8^ MutHER,^2^ and SUMMIT^3^).

## 2 METHODS

### 2.1 Patients and sample collection

The Geneplus cohort retrospectively enrolled 1184 invasive breast cancer patients from two hospitals who underwent circulating tumor DNA (ctDNA) analysis at the Geneplus‐Beijing Institute from March 2015 to September 2019. All enrolled patients were female breast cancer patients who underwent therapy at the Cancer Hospital, Chinese Academy of Medical Sciences, and Hunan Cancer Hospital and the Affiliated Cancer Hospital of Xiangya Medical School, Central South University. To understand the landscape of *TP53* mutation in the Chinese population and its correlation with clinical characteristics, we first analyzed *TP53* mutation in the Geneplus cohort.

To facilitate the study of *TP53* mutation and the efficacy of anti‐HER2 therapy according to the different HER2 amplification and mutation statuses, HER2 amplification‐positive was labeled as HER2+; HER2 amplification‐positive, mutation‐positive was labeled as HER2+/mut; HER2 amplification‐negative, mutation‐positive was labeled as HER2−/mut.

We used the MSK‐BREAST cohort to analyze the association between *TP53* mutation and the efficacy of anti‐HER2 antibody drugs. MSK‐BREAST data were downloaded from cBioPortal (http://www.cbioportal.org/). HER2+ metastatic breast cancer (MBC) patients who received anti‐HER2 antibody therapy (monotherapy or in combination with chemotherapy) were included in the analysis. The primary outcome measure was progression‐free survival (PFS).

To analyze the association between *TP53* mutation and the efficacy of HER2 TKIs in breast cancer patients, we combined ctDNA and prognosis data from phase Ib,^9^ phase Ic^10^ and phase II^11^ clinical studies of pyrotinib in HER2+ patients. HER2+ MBC patients who received HER2 TKIs (monotherapy or combined chemotherapy) in the MSK‐BREAST cohort were used to verify the prognostic value of *TP53* mutation and the efficacy of TKIs. The relevant information on each cohort can be found in the corresponding article. For the efficacy of HER2 TKIs in HER2−/mut breast cancer, a single‐arm, prospective, phase II study of pyrotinib in metastatic patients was performed (NCT03412383) at our center,^4^ and the MutHER cohort and SUMMIT cohort were obtained from the articles by Ma et al.^4^ and Hyman et al.^3^ to verify our findings. Since the SUMMIT study included patients with various types of cancer, we selected only breast cancer cases for verification.

**FIGURE 1:**
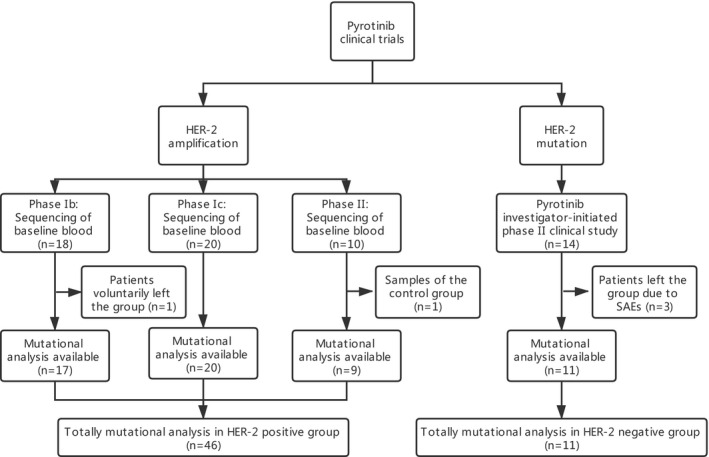
Study flowchart of the clinical trials of pyrotinib. HER2, human epidermal growth factor receptor 2; SAEs, Serious Adverse Events

All the studies were conducted in accordance with the Declaration of Helsinki and the principles of Good Clinical Practice and were approved by the Regulatory and Ethics Committees of Cancer Hospital, Chinese Academy of Medical Sciences. All participants provided written informed consent. The flowchart of the study is shown in Figure 1.

### 2.2 Sample collection and ctDNA analysis

Peripheral blood samples were collected from each patient using Streck tubes (Streck, Omaha, NE, USA) and centrifuged within 72 h to separate the plasma from peripheral blood cells. A panel of 1021 genes was assayed in the present study.^12^, ^13^ DNA extraction, library preparation, hybrid capture, sequencing, and analysis were performed as previously described.^12^, ^13^ Genomic DNA from lymphocytes was sequenced and used as the normal control sample.

### 2.3 Statistical analysis

The oncoplot and lollipop plots of amino acid changes and somatic interaction plots were generated using the maftools package.^14^ Pearson's *χ* ^2^ test was performed to compare categorical variables. PFS was calculated from the date of treatment initiation to the date of disease progression or death from any cause. Kaplan–Meier survival plots were generated, and curves were compared using log‐rank tests. All statistical tests were two‐sided, and *p* values below 0.05 were considered significant. The R package maftools was run in R software (version 3.6.0, Institute for Statistics and Mathematics, Vienna, Austria). Pearson's *χ* ^2^ test and independent samples t tests were performed with SPSS software (version 23, SPSS Inc., IBM). Kaplan–Meier survival plots were produced, and the number at risk was determined using MedCalc (version 19.5.3, MedCalc Software Bvba, Ostend, Flanders).

## 3 RESULTS

### 3.1 *TP53* mutation prevalence and patient characteristics

**FIGURE 2:**
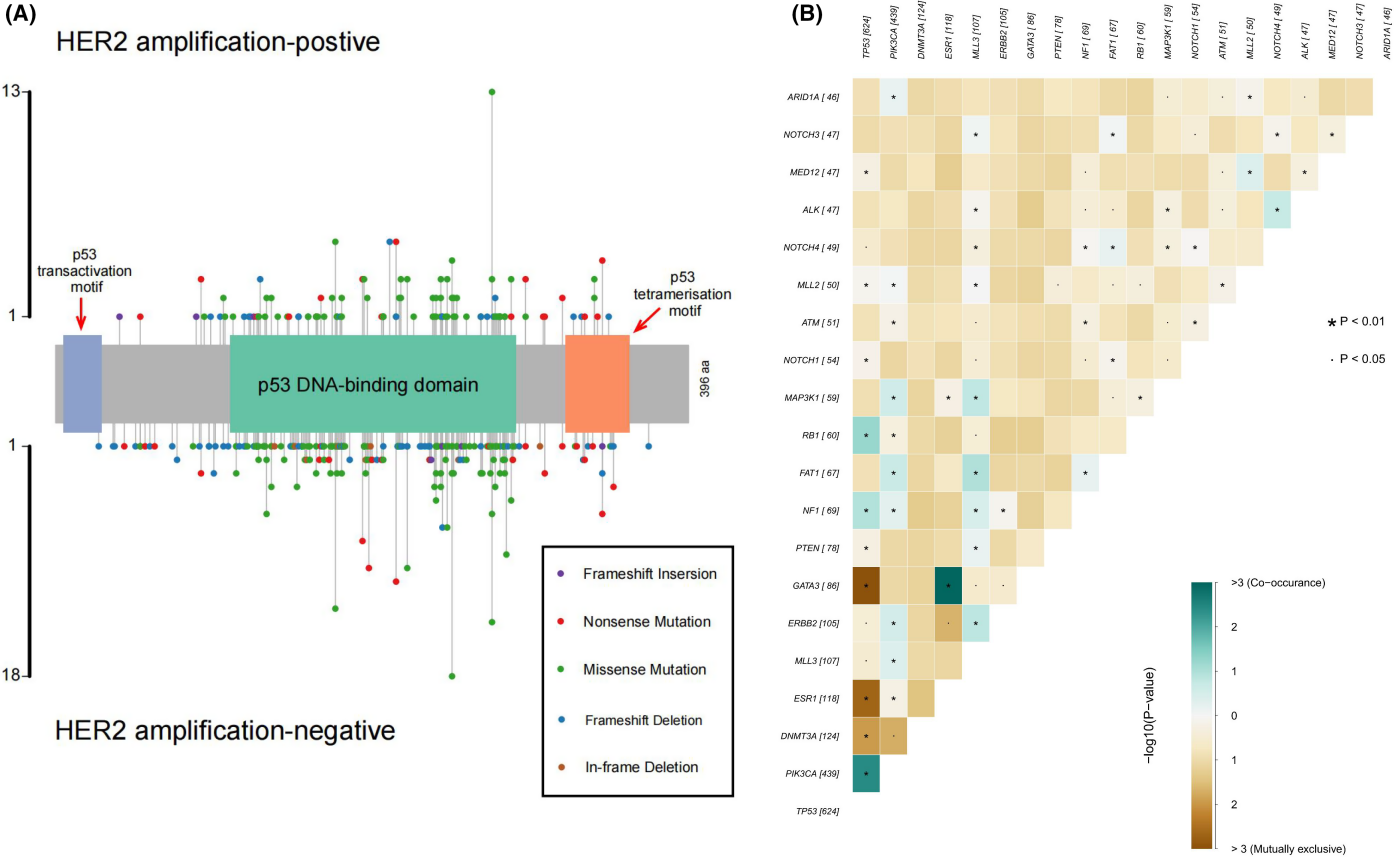
Lollipop and somatic interaction plots of *TP53*. (A) Lollipop plot of *TP53*. There was no obvious mutation hotspot in *TP53*; mutation sites were mostly concentrated in the DNA‐binding domain, accounting for 84.9% (567/668). There was no significant difference in mutation sites between HER2‐amplified and HER2‐nonamplified populations. NCBI Reference Sequence: NM_000546.5, *TP53* transcript variant 1. (B) Somatic interactions of *TP53* mutations. In the top 20 most common genes, *TP53* mutations co‐occurred with mutations in PIK3CA, RB1, and NF1 and were mutually exclusive with mutations in GATA3, ESR1, DNMT3A, MED12, PTEN, NOTCH4, ERBB2, and MLL3 (*p* < 0.05)

In total, 1184 metastatic breast cancer patients were enrolled in the Geneplus cohort. The clinical characteristics of the patients are summarized in Table 1. *TP53* mutations were detected in 53.1% (629/1184) of the patients. We identified 668 *TP53* somatic mutations at 314 different mutant sites (Table S1). A total of 73.6% (231/314) of the mutations were recorded in the COSMIC database (https://cancer.sanger.ac.uk/cosmic). There were 32 patients with multiple *TP53* mutations (32/1184, 2.7%), and the highest number of mutations was four. The mean variant allele frequency of *TP53* mutations was 17.24% and ranged from 0.03% to 86.34%. The most common type was missense mutations (58.5%, 391/668), followed by nonsense mutations (15.3%, 102/668), frameshift deletion mutations (14.7%, 98/668), splice site mutations (7.3%, 49/668), in‐frame deletions (2.2%, 15/668), frameshift insertion mutations (1.2%, 8/668), and splice region mutations (0.7%, 5/668). A lollipop diagram of the *TP53* mutations is presented in Figure 2‐A. *TP53* p. R273H (4.0%, 27/668), p. R248Q (3.3%, 22/668), p. R175H (2.7%, 18/668), and p. R213* (2.4%, 16/668) were the top four mutations ranked by frequency. A total of 84.9% (567/668) of *TP53* mutations occurred within the DNA‐binding domain (DBD, amino acids 102‐292, UniProtKB: P04637).

**TABLE 1:** Patient characteristics of the Geneplus cohort

Characteristics	No. of cases (%)			*p* value
	Total (*n* = 1184)	*TP53* wild type (*n* = 555)	*TP53* mutant (*n* = 629)	
Age at initial diagnosis				
≤35 years	170 (14.4)	71 (12.8)	99 (15.7)	0.149
>35 years	1014 (85.6)	484(87.2)	530 (84.3)	
Histopathological				
Invasive ductal carcinoma	1049 (88.6)	480 (86.5)	569 (90.5)	0.093
Invasive lobular carcinoma	45 (3.8)	24 (4.3)	21 (3.3)	
Other	90 (7.6)	51 (9.2)	39 (6.2)	
HR status				
Positive	696 (58.8)	395 (71.2)	301 (47.9)	<0.001
Negative	422 (35.6)	125 (22.5)	297 (47.2)	
Unknown	66 (5.6)	35 (6.3)	31 (4.9)	
HER2 status				
Positive	330 (27.9)	136 (24.5)	194 (30.8)	0.015
Negative	854 (72.1)	419 (75.5)	435 (69.2)	
Molecular subtype				
HR+/HER2‐	522 (44.1)	300 (54.1)	222 (35.3)	<0.001
HR+/HER2+	174 (14.7)	95 (17.1)	79 (12.6)	
HR‐/HER2+	155 (13.1)	41 (7.4)	114 (18.1)	
HR‐/HER2‐	267 (22.6)	84 (15.1)	183 (29.1)	
Unknown	66 (5.6)	35 (6.3)	31 (4.9)	

Abbreviations: HER2, human epidermal growth factor receptor 2; HR, hormone receptor.

The frequency of *TP53* mutation did show a certain relationship with clinical characteristics, and the differences in clinical characteristics between patients with and those without *TP53* mutations are shown in Table 1. HR‐positive patients had a lower mutation frequency than HR‐negative patients (43.2%, 301/696 vs. 70.4%, 297/422; *p* < 0.001), whereas HER2+ patients had a higher mutation frequency than HER2‐ patients (58.8%, 194/330 vs. 50.9%, 435/854; *p* = 0.015). The mutation rate of *TP53* also differed according to molecular subtype. The subtypes with the highest to lowest mutation frequencies were HER‐2‐positive breast cancer (114/155, 73.5%), triple‐negative breast cancer (TNBC, 183/267, 68.5%) and luminal‐subtype breast cancer (301/696, 43.2%) (*p* < 0.001).

### 3.2 *TP53* co‐occurring mutations

In 597 (94.9%) patients, *TP53* mutations were detected concurrently with mutations in other genes. The top five most frequent co‐occurring gene mutations were in PIK3CA (289/629, 46.0%), ERBB2 (69/629, 11.0%), MLL3 (67/629, 11.0%), NF1 (55/629, 8.7%) and PTEN (55/629, 8.6%). The top 10 gene mutations co‐occurring with *TP53* are shown in Figure S1. In the top 20 most common genes, *TP53* mutations co‐occurred with PIK3CA, RB1, and NF1 mutations (*p* < 0.01) and were mutually exclusive with GATA3, ESR1, DNMT3A, MED12, and PTEN mutations (*p* < 0.01) and NOTCH4, ERBB2, and MLL3 mutations (*p* < 0.05) (Figure 2B).

### 3.3 Effect of *TP53* mutation on the efficacy of monoclonal anti‐HER2 antibody drugs

In total, 425 treatment records from 188 patients were included from the MSK‐BREAST cohort.^8^ The characteristics of the 188 patients are listed in Table 2. Of the total treatment records included, 207 records (48.7%) showed that trastuzumab combined with chemotherapy was administered, 191 (44.9%) showed that trastuzumab plus pertuzumab combined with chemotherapy was administered, and 27 (6.3%) showed that trastuzumab monotherapy was administered. The median PFS for *TP53*‐mutant patients versus *TP53*‐wild‐type patients was 6.0 months versus 9.4 months [hazard ratio (HR) 1.42, 95% confidence interval (CI) 1.12–1.80, *p* = 0.004, Figure S2].

**TABLE 2:** Patient characteristics of the HER2‐positive cohort

Characteristics	HER2 amplification cohort				HER2 mutant cohort		
	No. of cases (%)				No. of cases (%)		
	Trastuzumab in the Geneplus cohort (*n* = 40)	Pyrotinib studies in HER2‐amplification patients (*n* = 46)^a^	Anti‐HER2 antibody drugs in the MSKCC‐BREAST cohort (*n* = 188)	Lapatinib in the MSKCC‐BREAST cohort (*n* = 45)	Pyrotinib investigator‐initiated clinical study (*n* = 11)	MutHER study (*n* = 14)^b^	Summit study (*n* = 25)
Age at initial diagnosis							
≤35 years	12 (30.0)	10 (21.7)	28 (14.9)	5 (11.1)	0 (0.0)	NA	NA
>35 years	28 (70.0)	36 (78.3)	160 (85.1)	40 (88.9)	11 (100.0)	NA	NA
Histopathology							
Invasive ductal carcinoma	33 (82.5)	42 (91.3)	151 (80.3)	33 (73.3)	11 (100.0)	NA	17 (68.0)
Invasive lobular carcinoma	1 (2.5)	0 (0.0)	17 (9.0)	4 (8.9)	0 (0.0)	NA	6 (24.0)
Other	6 (15.0)	4 (8.7)	20 (10.6)	8 (17.8)	0 (0.0)	0 (0.0)	2 (8.0)
HR status							
Positive	22 (55.0)	19 (41.3)	118	25 (55.6)	11 (100.0)	‐	‐
Negative	18 (45.0)	27 (58.7)	64	17 (37.8)	0 (0.0)	‐	‐
NA	0(0.0)	0 (0.0)	6 (3.2)	3 (6.7)	0 (0.0)	14 (100.0)	25 (100.0)
Visceral metastasis							
Yes	25 (62.5)	34 (73.9)	NA	NA	7 (63.6)	NA	NA
No	15 (37.5)	12 (26.1)	NA	NA	4 (36.4)	NA	NA
Pre anti‐HER2 treatment							
Yes	28 (70.0)	27 (58.7)	NA	NA	4 (36.4)	NA	NA
No	12 (30.0)	19 (41.3)	NA	NA	7 (63.6)	NA	NA
*TP53* status							
Positive	20 (50.0)	27 (58.7)	104 (55.3)	28 (62.2)	6 (54.5)	5 (35.7)	7 (28.0)
Negative	20 (50.0)	19 (41.3)	84 (44.7)	19 (37.8)	5 (45.5)	9 (64.3)	18 (72.0)

Abbreviations: HER2, human epidermal growth factor receptor 2; HR, hormone receptor; MSKCC, Memorial Sloan Kettering Cancer Center.

^a^ Including phase Ib, Ic, and II.

^b^ Since the clinical information given in the original text (16 patients) included 2 HER2 mutations of unknown significance, the author did not provide the mutation and clinical information of these two patients, so the clinical information of the 14 patients included in the mutation analysis could not be determined.

### 3.4 Effect of *TP53* mutation on the efficacy of anti‐HER2 TKIs in HER2 amplification‐positive patients

**FIGURE 3:**
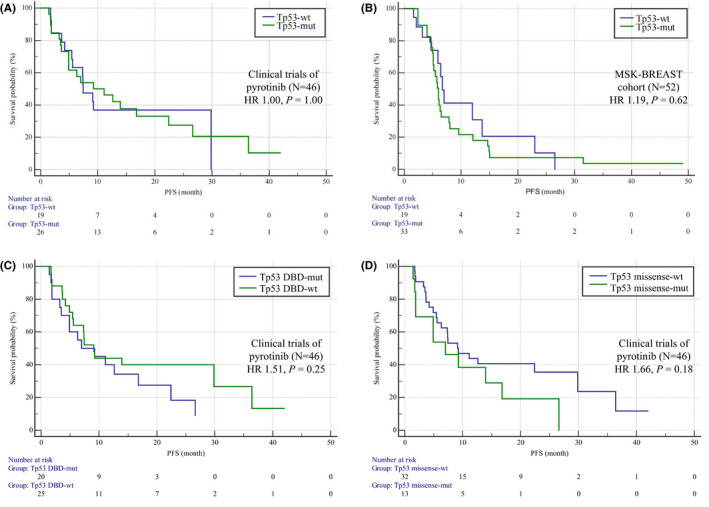
Kaplan–Meier curves of PFS for HER2 TKIs in HER2 amplification‐positive patients. (A) Comparison of PFS between patients with *TP53* mutations (*N* = 27) and *TP53*‐wild‐type patients (*N* = 19). HER2 amplification‐positive patients were assessed in phase Ib, phase Ic and phase II pyrotinib trials. There were no differences in PFS between the two groups (HR 1.00, 95% CI 0.49–2.04, *p* = 1.00). (B) Comparison of PFS between patients with *TP53* mutations (*N* = 33) and *TP53*‐wild‐type patients (*N* = 19) in the MSK‐BREAST cohort as validation, with the same result (HR 1.19, 95% CI 0.60–2.37, *p* = 0.62). (C) Comparison of PFS between patients with (*N* = 20) and without (*N* = 26) *TP53* DBD mutations in phase Ib, phase Ic, and phase II pyrotinib trials. No difference in PFS was shown between the two groups (HR 1.51, 95% CI 0.74–3.05, *p* = 0.25). (D) Comparison of PFS between patients with (*N* = 14) and without (*N* = 32) *TP53* missense mutations in phase Ib, phase Ic, and phase II pyrotinib trials. No difference in PFS was shown between the two groups (HR 1.66, 95% CI 0.80–3.48, *p* = 0.18). HR, hazard ratio. PFS, progression‐free survival. HER2, human epidermal growth factor receptor 2. DBD, DNA‐binding domain; Mut, mutation; Wt, wild type

To determine whether *TP53* mutation has predictive value in HER2+ breast cancer patients, we first analyzed ctDNA sequencing results and the efficacy of pyrotinib in phase Ib, phase Ic, and phase II clinical trials led by our center. The characteristics of the cohorts from the pyrotinib clinical trials are listed in Table 2. In the phase Ib, Ic, and II clinical trials, ctDNA sequencing results were available for 46 patients; 27 *TP53* mutations were detected. Overall, we found that mutation of *TP53* did not predict the efficacy of pyrotinib; the median PFS for patients with *TP53* mutation versus that of patients with wild‐type *TP53* was 9.2 months versus 7.4 months (HR 1.00, 95% CI 0.49–2.04, *p* = 1.00, Figure 3A). We then included patients from the MSK‐BREAST cohort for validation. Fifty‐two available treatment records from 45 patients were analyzed. The characteristics of the 45 patients are listed in Table 2. The median PFS for the patients with mutant *TP53* versus that of the patients with wild‐type *TP53* was 6.0 months versus 6.7 months (HR 1.19, 95% CI 0.60–2.37, *p* = 0.62, Figure 3B).

We refined the *TP53*‐mutated population in an attempt to more precisely predict the efficacy of pyrotinib and found that mutations in the DBD of *TP53* did not predict its efficacy (HR 1.51, 95% CI 0.74–3.05, *p* = 0.25, Figure 3C). Nonetheless, *TP53* missense mutations tended to be associated with shorter PFS than other *TP53* mutations and wild‐type *TP53* (HR 1.66, 95% CI 0.80–3.48, *p* = 0.18, Figure 3D).

### 3.5 *HER2* mutation in HER2 amplification‐positive patients

Before analyzing the gene markers for their ability to predict the efficacy of HER2 TKIs in HER2−/mut patients, we first analyzed the HER2 TKIs in HER2+/mut patients to understand the impact of HER2 mutation on anti‐HER2 treatment. In phase Ib, phase Ic and phase II pyrotinib clinical trials, eight of 46 (17.4%) patients had HER2 mutations. The median PFS of HER2‐mutant patients was 11.1 months, which was shorter than the 4.9 months of HER2 wild‐type patients (HR 2.12, 95% CI 0.90–5.01, *p* = 0.09, Figure S3A).

We further analyzed the PFS of patients with *HER2* and *TP53* comutations, and the results showed no difference in PFS between patients with *TP53* and *HER2* comutation (Tp53‐mut/HER2‐mut), patients with *TP53* mutation and wild‐type *HER2* (Tp53‐mut/HER2‐wt), and patients with wild‐type *TP53* (Tp53‐wt) (reference: Tp53‐wt; Tp53‐mut/HER2‐mut: HR 1.70, 95% CI 0.55–5.24, *p* = 0.36; Tp53‐mut/HER2‐wt: HR 0.90, 95% CI 0.43–1.91, *p* = 0.79, Figure S3B).

### 3.6 Effect of *TP53* on the efficacy of anti‐HER2 TKIs in HER2−/mut patients

**FIGURE 4:**
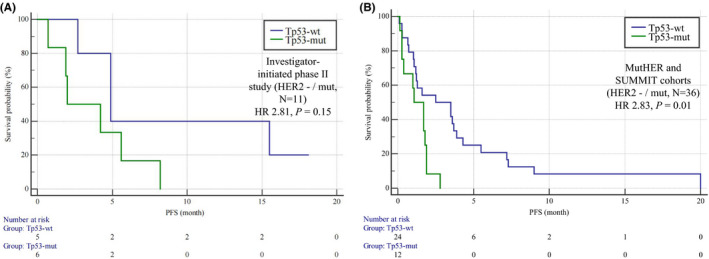
Kaplan–Meier curves of PFS for HER2 TKIs in HER2 mutation‐positive amplification‐negative patients (HER2−/mut). (A) Comparison of PFS between patients with (*N* = 6) and without (*N* = 5) *TP53* mutations in our investigator‐initiated phase II study. Patients with *TP53* mutations showed a trend of poor efficacy in response to pyrotinib (HR 2.81, 95% CI 0.69–11.43, *p* = 0.15). (B) Comparison of PFS between patients with (*N* = 12) and without (*N* = 24) *TP53* mutations in the combination analysis of the MutHER and SUMMIT cohorts. Patients with *TP53* mutations showed a trend toward poor efficacy in response to pyrotinib (HR 2.83, 95% CI 1.25–6.43, *p* = 0.01). HR, hazard ratio. PFS, progression‐free survival. HER2, human epidermal growth factor receptor 2. Mut, mutation; Wt, wild type

First, we analyzed the correlation between *TP53* mutation and treatment efficacy based on the results of a prospective phase II clinical study of pyrotinib in HER2−/mut breast cancer patients conducted at our center.^4^ As of November 1, 2019, 11 patients had been enrolled in the study. Patients with *TP53* mutations showed a trend toward a shorter PFS with pyrotinib, but due to the small sample size, the difference was not statistically significant. The median PFS for patients with mutant *TP53* versus that of patients with wild‐type *TP53* was 4.9 months versus 2.0 months (HR 2.81, 95% CI 0.69–11.43, *p* = 0.15, Figure 4‐A).

To further test our hypothesis, two clinical trials of neratinib (irreversible pan‐HER TKIs such as pyrotinib), the MutHER study^2^ and the SUMMIT study,^3^ were analyzed. A total of 36 HER2−/mut patients met the inclusion criteria, including 14 from the MutHER cohort and 22 from the SUMMIT cohort. We found that *TP53* mutation clearly predicted the efficacy of HER2 TKIs. The median PFS of patients with wild‐type *TP53* was 2.5 months, whereas that of patients with mutant *TP53* was only 1.1 months (HR 2.83, 95% CI 1.25–6.43, *p* = 0.01, Figure 4B).

## 4 DISCUSSION

*TP53* mutation has attracted widespread attention because it can increase chromosome instability, leading to effects such as increased oncogene amplification, deep deletion of tumor suppressor genes and, ultimately, a poor prognosis.^15^ *TP53* mutation is the most common mutation in breast cancer, with an incidence of approximately 50% in unselected breast cancer patients in our study, which is higher than that reported in other studies (approximately 30%–40%^7^, ^15^). Multiple studies have confirmed that the mutation frequency of *TP53* differs among molecular subtypes.^7^ The mutation rate of *TP53* in HER2‐positive subtypes has been reported to be as high as 70%, which is consistent with our own research. Therefore, the higher frequency of *TP53* mutations in our data may be because of the different molecular subtypes of the patients included, although ethnic differences in the Chinese population may have also played a role.

*TP53* mutations often lead to early‐onset breast cancer and predict a poor prognosis.^7^ However, whether *TP53* can predict the efficacy of antitumor therapy is still controversial. Some authors have posited that *TP53* can predict the efficacy of anthracycline‐ or taxane‐based chemotherapy regimens.^5^ Because of the controversy over the predictive role of *TP53*, this gene has not yet been used as a biomarker for the management of breast cancer.^6^ In our study, *TP53* was able to predict the efficacy of anti‐HER2 antibody‐based drugs and the efficacy of HER2 TKIs in HER2−/mut patients.

Different mutation types and mutation sites in *TP53* are related to the therapeutic effect of TKIs, which has been reported in other articles. Epidermal growth factor receptor (EGFR), also known as human epidermal growth factor receptor 1 (HER1), is a member of the HER family. Although relationships between EGFR TKIs and *TP53* mutations have been well studied, the results are controversial. Most articles have found that patients with *TP53* mutations have a lower disease control rate (DCR), shorter PFS and even overall survival (OS) for a variety of EGFR TKIs than those without *TP53* mutations.^16^, ^17^, ^18^, ^19^, ^20^ Labbé et al.^19^ found that *TP53* missense mutations were better predictors of the efficacy of EGFR TKIs than all *TP53* mutations in lung cancer patients, as those with *TP53* missense mutations had marginally lower response rates and shorter PFS on EGFR TKI therapy than those without *TP53* missense mutations.

Functionally, mutations in *TP53* can result in loss of its tumor‐suppressive properties and gain of oncogenic activity, especially when missense mutations occur in the DBD.^19^ Patients with mutations in exons 5–8 (encoding the DBD region of the p53 protein) who are treated with EGFR TKIs were found to have a worse prognosis than wild‐type *TP53*‐harboring controls.^16^ Canale et al. found that exon 8 mutation and exon 19 deletion were also associated with significantly lower DCR and shorter PFS and OS than other mutations.^17^, ^22^ Nevertheless, such relationships between *TP53* DBD mutations and treatment efficacy were not found in our research. One possible reason is because the mechanisms of resistance to HER2 TKIs and resistance to EGFR TKIs are different. Given that *TP53* had different predictive value in patients with different HER2 statuses in this study, we believe that the utility of *TP53* for predicting efficacy is heterogeneous and population‐specific and therefore cannot be generalized to all populations of patients with breast cancer.

The possible underlying mechanism through which *TP53* mutation hinders the efficacy of monoclonal anti‐HER2 antibody drugs is by augmenting mitogen‐activated protein kinase (MAPK) and phosphatidylinositol 3‐kinase (PI3K) signaling through enhanced recycling and/or stability of ERBB2/EGFR; this effect leads to transcriptional phosphoactivation of heat shock transcription factor‐1 (HSF1), the target of which is the chaperone Hsp90, which in turn stabilizes Her2 and mut*P53* itself at the protein level.^23^, ^24^ However, pan‐HER TKIs inhibit both Her2 and EGFR, thus suppressing the Her2‐HSF1‐mut*P53* interaction and leading to destabilization of the mut*P53* protein in cancer cells.^7^, ^24^ This mechanism may explain why HER2 TKIs can overcome anti‐HER2 resistance in *TP53* mutation‐positive patients. Nonetheless, there is no explanation for why HER2 TKIs cannot overcome the resistance induced by *TP53* mutation in HER2−/mut patients. According to a previous study, we speculate that in HER2 mutation‐positive patients, in addition to *TP53* mutation, the Her2‐HSF1‐mut*P53* feed‐forward loop is likely to be reactivated regardless of HER2 TKIs, leading to HER2 TKI resistance.

The results of this study offer suggestions for clinical treatment. In HER2+ patients, *TP53* mutations reduce the efficacy of anti‐HER2 antibody drugs but do not affect that of HER2 TKIs. Therefore, antibody treatment combined with TKI therapy may improve the therapeutic efficacy. If HER2−/mut patients carry *TP53* mutations, treatment with HER2 TKIs alone should be avoided, and combined chemotherapy should be adopted to improve the treatment response.

For MBC patients, the use of ctDNA for NGS detection and to optimize the treatment of breast cancer has been increasingly recognized. Just as ERBB2, PI3KCA, ESR1, and other genes have guided the selection of breast cancer treatment strategies, *TP53* mutations, with their high mutation rate and value in predicting anti‐HER2 treatment response, will certainly play a role in precision therapy in the future and become very valuable therapeutic biomarkers in clinical practice.^4^, ^25^, ^26^

In contrast to the many studies on its role as a biomarker, there has been little work to date on exploiting the mutant protein as a target to treat breast cancer. In addition, there are still no approved agents that specifically target *TP53* mutations. Scientists have studied several ways of restoring the function of p53 in cancer cells. These include reactivating mut*P53* to the wild‐type form, eliminating mut*P53*, blocking the negative regulators' mouse double minute 2 homolog (*MDM2*) and *MDM4*, gene therapy with vectors containing wild‐type p53, identifying synthetic lethal partners of mut*P53*, and treatment with compounds that promote early termination codon interpretation.^21^ However, only a few drugs have entered clinical trials. APR‐246 was the first mut*P53*‐reactivating compound that progressed to clinical trials because it appeared to revert at least some of the mutant conformations of p53 to their wild‐type form.^7^, ^21^ There are no final results from these clinical trials as of yet. Unlike APR‐246, thiosemicarbazone (COTI‐2) appears to act both by reactivating mut*P53* and inhibiting the PI3K/serine–threonine kinase (Akt)/mammalian target of rapamycin (mTOR) pathway. Currently, COTI‐2 is undergoing evaluation for the treatment of gynecological cancers in a phase I clinical trial (NCT02433626).

In addition to directly targeting mut*P53*, there are some other promising findings that can be further explored. WEE1 is a tyrosine kinase that regulates cell cycle progression by governing the G2 checkpoint, a proven strategy to eliminate G2 cell cycle arrest and to utilize G1 checkpoint deficiency in p53‐deficient tumor cells, thereby enhancing their apoptotic response to DNA damage.^27^ Both phase I and phase II clinical trials have suggested that AZD1775, a Wee‐1 inhibitor, as monotherapy or in combination with chemotherapy has high efficacy in treating different cancers, with a well‐tolerated cytotoxic profile.^27^, ^28^ Although there has been no large‐scale clinical research in breast cancer, research from Sand et al. highlights the potential clinical utility of AZD1775 for overcoming trastuzumab resistance in HER2‐positive breast cancer,^29^ providing new evidence to support the application of AZD1775 in breast cancer.

There are some limitations to our study. First, there was a relatively small number of samples available for the exploration of the predictive value of *TP53* in HER2‐TKIs in HER2+ and HER2−/mut patients. This small number of samples is due to the relatively low frequency of HER2 mutation, and the sample enrolled in our studies was from phase Ib, Ic, and II clinical studies of pyrotinib, so the samples cannot be expanded. Regardless, our conclusions are based on our own clinical study data and were verified with data from a completely independent public database, which reflects the reliability of the conclusions. Second, because we used the MSKCC‐Breast cohort, SUMMIT cohort, and MutHER study cohort to verify the results, some clinical information was incomplete (e.g., the clinicopathological information of the MutHER cohort could not be obtained). In addition, the number of the study cohort was relatively small, so it was difficult to conduct multivariate Cox analysis on the data. Another limitation is that the medication information of the MSK‐BREAST cohort had not been updated in time for inclusion in this study. It is possible that the results of this study will change as the follow‐up times are updated in the cohort. To truly understand the clinical value of *TP53* in predicting the efficacy of anti‐HER2 antibodies and HER2‐TKIs in breast cancer patients, a large, prospective clinical study should be designed to verify the results reported herein.

In summary, *TP53* mutations were detected in almost half of the included breast cancer patients. Patients who were HR negative and HER2 amplification positive had a higher *TP53* mutation frequency than those who were HR positive and HER2 amplification negative. Patients carrying *TP53* mutation had shorter PFS in response to anti‐HER2 antibody treatment than those without *TP53* mutation. Overall, the value of *TP53* mutation in predicting HER2 TKI efficacy remains controversial, and a poor prognosis in response to HER2 TKIs may only be seen in HER2−/mut patients and not in HER2+ patients.

## ETHICAL APPROVAL

The collection and sequencing of peripheral blood samples of patients who consented to participate in the ctDNA analysis were approved by the Institutional Review Boards of Cancer Hospital, Chinese Academy of Medical Sciences (Approval No: 16‐038/1117). Written informed consent was obtained from all patients whose samples were used in the ctDNA analysis.

## CONFLICT OF INTEREST

The authors declare that there are no competing interests to disclose.

## AUTHOR CONTRIBUTIONS

Binliang Liu, Zongbi Yi, Binghe Xu, and Fei Ma carried out study concept and design. Binliang Liu and Yanfang Guan were involved in performance of experiments. Binliang Liu, Zongbi Yi, Yanfang Guan, Binghe Xu, and Fei Ma carried out acquisition analysis and interpretation of data. Binliang Liu, Zongbi Yi, and Fei Ma contributed to the manuscript preparation. All authors were involved in editing and reviewing of the manuscript.

## Supporting information


Figure S1

Figure S2

Figure S3

Table S1

## Data Availability

The data of Geneplus cohort are available from the corresponding author upon reasonable request. The MSK‐BREAST data were downloaded from cBioPortal (http://www.cbioportal.org/). The clinical data and part of sequencing data of phase Ib (DOI: 10.1200/JCO.2016.69.6179), phase Ic (DOI: 10.1158/1078‐0432.CCR‐18‐4173) and phase II (DOI: 10.1200/JCO.19.00108) clinical trials of pyrotinib and an investigator‐initiated phase II study of pyrotinib (DOI:10.1038/s41523‐020‐00201‐9) are available in their respective articles, detailed sequencing results of pyrotinib clinical trials are available from the corresponding author upon reasonable request. The clinical and sequencing information for the MutHER cohort and SUMMIT cohort are openly available in articles by Ma et al. ( DOI: 10.1158/1078‐0432.CCR‐17‐0900) and Hyman et al. (DOI: 10.1038/nature25475).
