# Supplementary figures and images for: Molecular landscape of TP53 mutations in breast cancer and their utility for predicting the response to HER‐targeted therapy in HER2 amplification‐positive and HER2 mutation‐positive amplification‐negative patients
Source: Cancer Med. 2022 Apr 7;11(14):2767–78. doi: 10.1002/cam4.4652 (PMC9302303; doi:10.1002/cam4.4652)

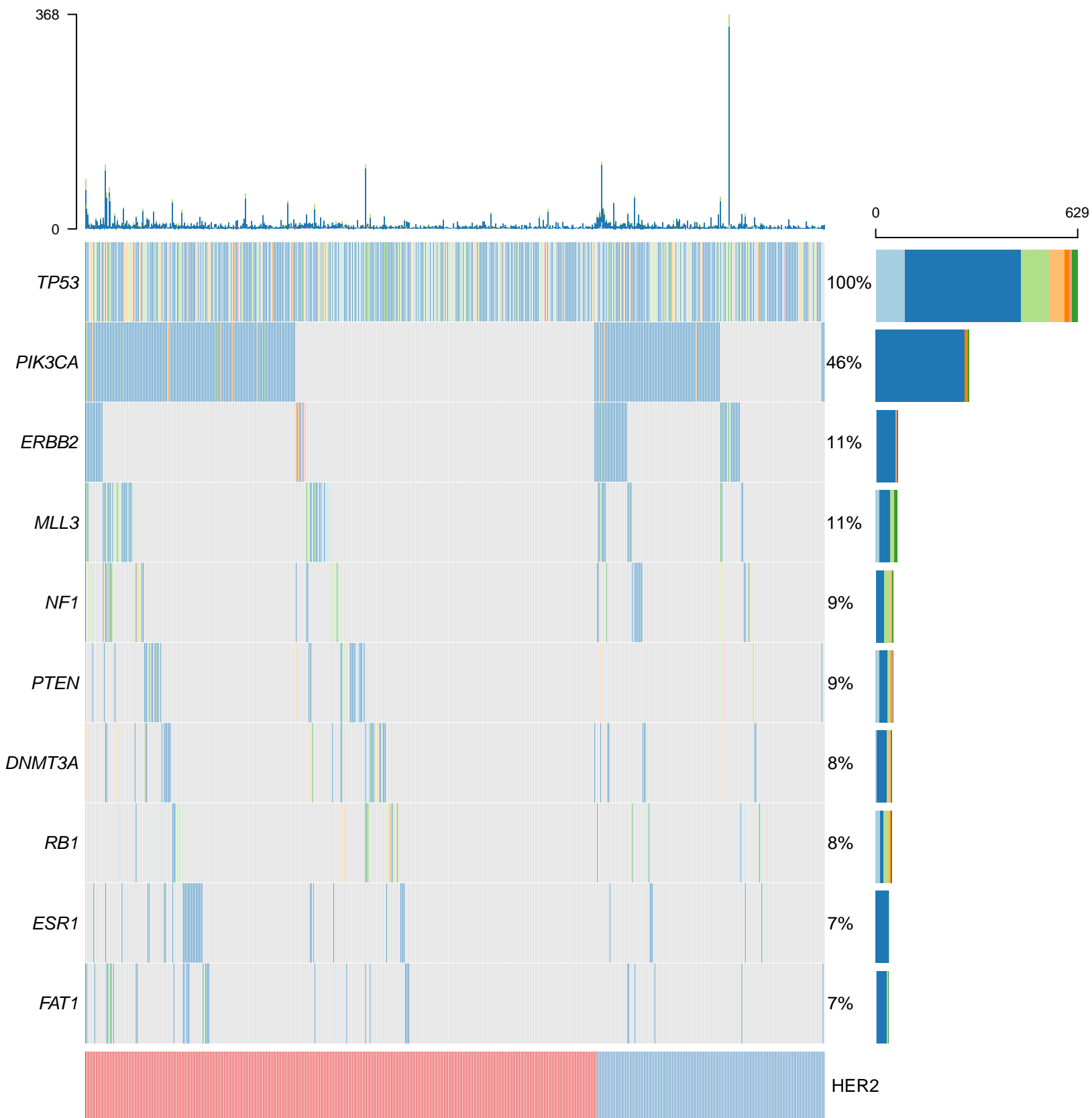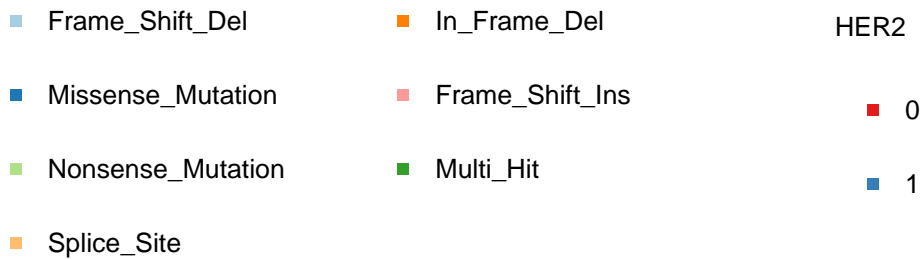

Supplement: Supplementary file 1 — Figure S1 [file CAM4-11-2767-s003.pdf]

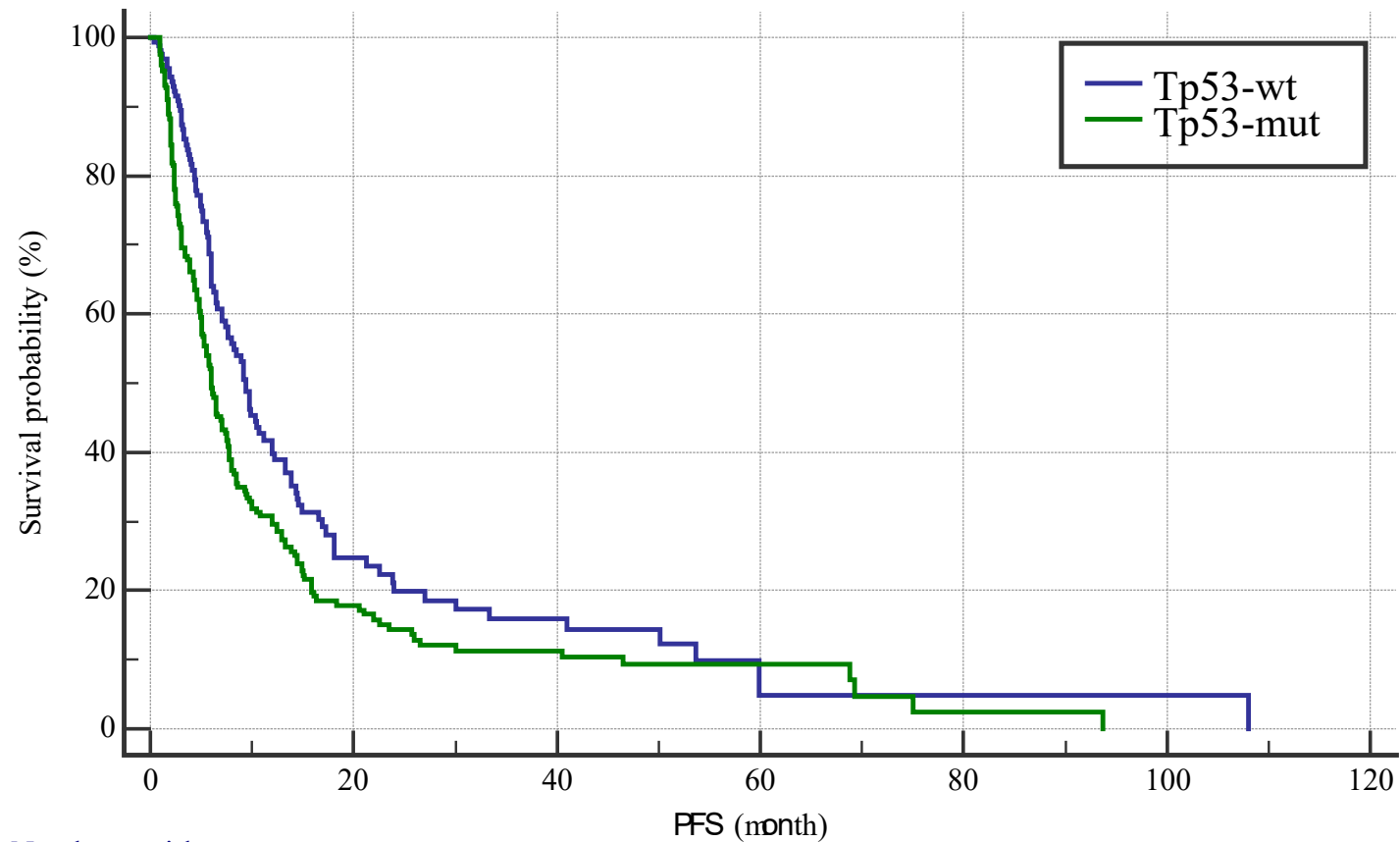

Number at risk

Group: Tp53-wt

167

21

10

1

1

1

0

Group: Tp53-mut

257

26

12

4

1

0

0

Supplement: Supplementary file 2 — Figure S2 [file CAM4-11-2767-s002.pdf]
