# Supplementary material for: Molecular landscape of TP53 mutations in breast cancer and their utility for predicting the response to HER‐targeted therapy in HER2 amplification‐positive and HER2 mutation‐positive amplification‐negative patients
Source: Cancer Med. 2022 Apr 7;11(14):2767–78. doi: 10.1002/cam4.4652 (PMC9302303; doi:10.1002/cam4.4652)
Supplement: Supplementary file 3 — Figure S3 [file CAM4-11-2767-s004.pdf]

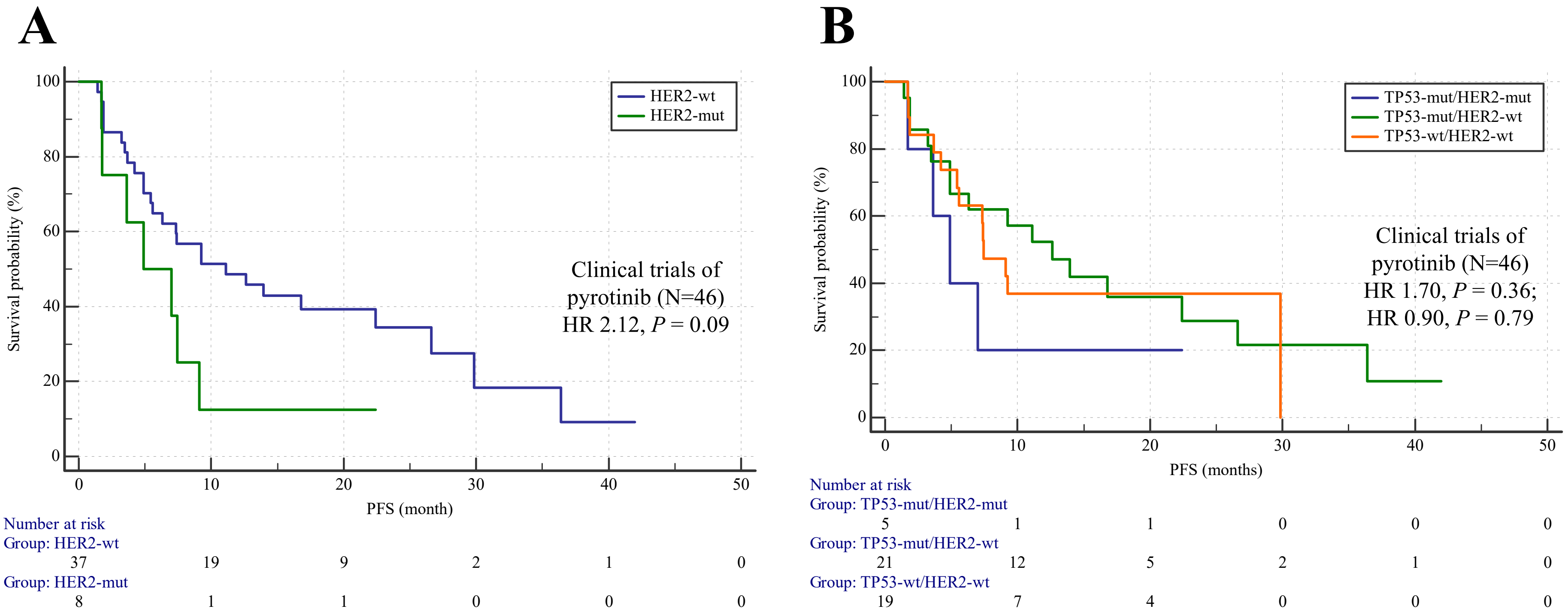
A
Survival probability (%)
100
80
60
40
20
0
0
10
20
30
40
50
PFS (month)
HER2-wt
HER2-mut
Clinical trials of
pyrotinib (N=46)
HR 2.12, *P* = 0.09
Number at risk
Group: HER2-wt
37 19 9 2 1 0
Group: HER2-mut
8 1 1 0 0 0
B
Survival probability (%)
100
80
60
40
20
0
0
10
20
30
40
50
PFS (months)
TP53-mut/HER2-mut
TP53-mut/HER2-wt
TP53-wt/HER2-wt
Clinical trials of
pyrotinib (N=46)
HR 1.70, *P* = 0.36;
HR 0.90, *P* = 0.79
Number at risk
Group: TP53-mut/HER2-mut
5 1 1 0 0 0
Group: TP53-mut/HER2-wt
21 12 5 2 1 0
Group: TP53-wt/HER2-wt
19 7 4 0 0 0
